# Promoting Good Nonhuman Primate Welfare outside Regular Working Hours

**DOI:** 10.3390/ani13081423

**Published:** 2023-04-21

**Authors:** Sabrina Brando, Augusto Vitale, Madison Bacon

**Affiliations:** 1 AnimalConcepts, Teulada, P.O. Box 378, 03725 Alicante, Spain; 2 Center for Behavioural Sciences and Mental Health, Istituto Superiore di Sanità, 00161 Rome, Italy; 3 Department of Animal Science, University of Minnesota-Twin Cities, Saint Paul, MN 55455, USA

**Keywords:** animal welfare, captivity, environmental enrichment, primates, zoo, 24/7, off hours, back of house, night-time

## Abstract

**Simple Summary:**

The responsibility to provide a complex environment and environmental enrichment is an essential element of primate wellbeing programs that should be approached from a 24-h perspective and planned according to the species and individual needs. It is necessary to be aware that animals’ care needs may vary over a 24-h period, and the necessary provisions for good welfare at night and out-of-office hours when care staff are not present may not be the same as during the day. The present review presents a case for increased attention to the welfare of non-human primates during night hours when care staff are not on site, including relevant topics concerning the care of animals during these hours and the use of related technologies to both assess and facilitate wellbeing overnight.

**Abstract:**

Promoting good primate welfare outside of daylight hours is an important task. The responsibility to provide a complex environment and environmental enrichment is an essential element of primate wellbeing programs that should be approached from a 24-h perspective and planned according to the species and individual needs, including giving animals the ability to interact with and control their environment during hours when animal care staff are not present. One must be aware, however, that their needs may differ at night-time from their care needs during the day when staff are present. Assessing welfare and providing enrichment during times when staff are not on hand can be achieved through the use of a variety of technologies, such as night-view cameras, animal-centred technologies, and data loggers. This paper will address the relevant topics concerning the care and welfare of primates during off-hours, and the use of related technologies to facilitate and assess wellbeing at these times.

## 1. To Promote Good Animal Welfare outside Regular Working Hours

Providing complex and enriched environments that offer choice and control is a core building block of professional primate care. This is indicated and enshrined in law in several jurisdictions: in the EU, for example, Directive 2010/63/EU on the protection of animals used in scientific procedures states in Annex III, “All animals shall be provided with space of sufficient complexity to allow expression of a wide range of normal behaviour,” and “they shall be given a degree of control and choice over their environment to reduce stress-induced behaviour” [1].

Animal care and welfare programs have made advances in recent decades, facilitated through research and the implementation of best practices in, for example, veterinary care, nutrition, environmental enrichment, habitat design, and animal training. However, animal welfare at night and other hours when staff are absent or only a few on duty, as when the institution is closed (henceforth referred to as off-hours) is not usually discussed. Addressing off-hours welfare requires that we are aware that animals may have different welfare needs at night and that they require and deserve the same level of choice, complexity, and control of their environment during these hours as during the day. Furthermore, the technologies and interventions that provide choice and control during the day may not be the same as the ones necessary at night based on species-specific nocturnal behaviours and needs.

Animal care and welfare must be considered at all times of day, all days of the week, and throughout the animal’s life (Brando & Buchanan-Smith, 2018 [2]). Humans, like most primates, are diurnal, or active during the day (Ferrante et al., 2015 [3]). Thus, humans involved in animal welfare tend to focus on changes that are made during daylight hours. In writing this paper, we originally set out to outline a primate welfare program taking a 24-h approach (Brando & Buchanan-Smith, 2018 [2]). However, we found that while the night-time behaviours of primates living in human care are described in the literature (Fruth et al., 2018 [4]), practical evidence and discussion on how to apply this information to optimize primate welfare during off-hours is still a relatively unexplored area, particularly for nocturnal species. Given this lack of literature, we have opted to include personal observations in our discussion and to highlight examples from other primate species to illustrate the topic. In addition to sharing personal observations and discussing the literature on the night-time care of primates, we propose several refinements to the practical care of primates that we believe will enhance animal welfare during off-hours.

We will discuss the concept of off-hours, inspired by the 24/7-across-lifespan approach and the 14-point framework described by Brando and Buchanan-Smith (2018) [2], with particular attention to the back-of-house concept, also referred to as off-exhibit, a concept adapted and translated worldwide, considering the wide range of areas and spaces where animals may be closed in overnight or at other times (Brando & Coe, 2022 [5]). In this context, we will discuss animal welfare and the challenges of caring for animals during off-hours, resulting in, for example, variations in the quality of care, all the time that environmental enrichment, species differences, sleep and other night-time behaviour, safety and monitoring, and the use of technology are addressed. We will conclude with some of the possible technologies that can be used to monitor and assess animal welfare during off-hours and the need to provide complex areas that allow for choice, control, and increased levels of agency at all times of day.

Although our focus is on animals housed in zoological parks (henceforth zoos), we recognise that many non-human primates are housed in other settings such as research laboratories and sanctuaries and suggest that many of these concepts could also be applied in such contexts. A professional animal welfare program, no matter the facility, should cover aspects such as, for example, the complexity of the environment, social structure, feeding, resting, and sleeping opportunities, safety, and the human-animal relationship, among other relevant topics. Characteristics of the individual (e.g., sex, age, and species) should also be taken into consideration to optimize their wellbeing [6].

## 2. Animal Welfare and Wellbeing at Night

The terms “animal welfare” and “animal wellbeing” have both been used interchangeably over the years (Moberg & Mench, 2000 [7]) to describe the state of the animal. Animal welfare science and practice focuses on the welfare of individuals (Brando & Buchanan-Smith, 2018 [2]), and definitions and methods of measuring animal welfare are regularly discussed (e.g., Fraser, 2009 [8]; Mason & Mendl, 1993 [9]; Veasey, 2017 [10]). Animal wellbeing revolves around the perceptions, feelings, and experiences of the animal themselves. Animal wellbeing comprises the psychological and physical experience, including all needs and preferences, of an individual animal as perceived by herself or himself, and the ability to exert agency over one’s own life to a meaningful extent. A wide variety of experiences, ranging from positive to negative, affect animal wellbeing; a variety of inputs may all act at the same time and may be synergistic rather than simply additive in their consequences. Therefore, the evaluation of wellbeing should consider all measurable factors and how they interact. The focus should be on monitoring and assessing animals based on their experiences, and on promoting agency, choice, and control, and, predominantly, positive wellbeing (Brando & Coe, 2022 [5]).

People have long been aware that an animal’s welfare in captivity is affected by their environment, nutrition, housing, handling, and social structure (Olsson & Westlund, 2007 [11]), as well as interactions with humans (Hemsworth and Coleman, 2010 [12]; Hill & Broom, 2009 [13]; Whitham & Wielebnowski, 2013 [14]). Animal welfare science has evolved to cover these topics and introduce others, including animal sleep, transport, enrichment, and human-animal interactions. Various models and frameworks for considering and measuring animal welfare have been introduced and discussed over the years, such as the three philosophies of natural living, basic health, and affective states identified by Fraser et al. (1997) [15]. More recently, the five domains concept of animal welfare has been proposed, which entails the possibility of actively promoting positive feelings and wellbeing, rather than just avoiding negative states, in areas of interest such as nutrition, physical environment, health, and behaviour (Mellor et al., 2020 [16]). The 24/7 across-lifespan approach, adapted from 12 criteria into 14 for welfare and initially developed for farm animals (Welfare Quality, 2009), gives additional focus to affective states and species-specific social behaviour to adapt these points for use with captive wild animals (Brando & Buchanan-Smith, 2018 [2]). These 14 points (see page 2) encourage animal caretakers to consider all aspects of an animal’s life throughout their lives, including the off-hours (Table 1). This framework can be also applied to primates housed in research laboratories, considering certain aspects of the care, such as the spatial, structural, and procedural limitations that, in general, are still more significant than among primates housed in zoos and sanctuaries.

## 3. Rest and Sleep with Primates

### 3.1. Sleep and Night-Time Behaviour

The availability of appropriate and comfortable hiding areas as safe locations for resting and sleeping is important for nonhuman primates in human care. Adequate sleep is essential for good welfare; a recent study of zoo-housed chimpanzees, for example, found that disruptions in sleep led to negative behavioural outcomes such as increased inactivity and self-directed behaviours (Ayuso et al., 2023). It is well-established that sleep deprivation has negative behavioural and physiological consequences for humans ([48]; Bechtol et al., 2010 [49]), and the same is likely to be true for other animals, including nonhuman primates [50]; Fruth et al., 2018 [4]). Sleep disturbances, such as overnight zoo events, have been observed to impact the behaviour of primates by increasing their activity levels (Proctor & BastianSmurl, 2020 [51]) and their level of abnormal behaviour (Bastian et al., 2020 [52]), highlighting the need for adequate sleeping areas where animals can feel comfortable and undisturbed at rest (Brando & Buchanan-Smith, 2018 [2]). Wild baboons were also recorded as sacrificing sleep in favour of vigilant behaviours when they are in unfamiliar environments, and they do not compensate for a lack of sleep through deeper sleep (Loftus et al., 2022 [53]). In contexts where primates are under human care, it can be surmised that disturbances which increase vigilance will have a similar impact on sleep deprivation.

Furthermore, we have a growing understanding of the sleeping environments preferred by different primate species. Different species of primates have different needs, and further, groups and individuals can have different needs and preferences when it comes to sleeping sites as well. In the wild environment, sleep site preference may be motivated by factors such as safety and nearby resources (Markham et al., 2016 [54]), which may also motivate primates living in captivity to some extent. These species and individual differences highlight the need for providing different nesting options that reflect, for example, seasonal preferences for resources such as sources of warmth; changes in weather or climate and conditions like cold, wind, and heat also affect night-time behaviours (Reinhardt et al., 2019 [55]). Quiet areas away from the public or activities should also be considered for nocturnal animals so they may sleep undisturbed during the day.

Caine et al. (1992) [56] found that red-bellied tamarins (*Saguinus labiatus*) consistently chose a sleeping nest-box that offered maximum concealment, while boxes that offered moderate or minimum concealment were less frequently chosen; the tamarins also selected the sleeping box that was the maximum distance from the ground. When presented with only the nest box that offered minimum concealment, tamarins increased their levels of visual scanning prior to entering the nest box, highlighting the importance of providing safe hiding places (Caine et al., 1992 [56]). One researcher noted the same behaviour in a captive colony of common marmosets (*Callithrix jacchus*) housed in a research laboratory (Augusto Vitale, pers. obs., 1995). Knowing as much as possible about the species, individual needs, and preferences of the animal allows primate caregivers to enhance welfare by providing an appropriate number and style of resting and sleeping places, such as nest boxes, and understanding that animals should have the choice to come and go from these places as a safe haven without being trapped.

Great apes in the wild construct elaborate nests out of sight of predators in trees (Goodall, 1962 [23]; Fruth et al., 2018 [4]). In many facilities that house these species, great apes are provided with hammocks, raised sleeping platforms, and nesting material so that they can choose their preferred sleeping space. Great apes’ ability to construct high-quality nests is considered essential to good quality sleep and achieving the positive effects of sleep on cognition (Fruth et al., 2018 [4]). There are, however, species-specific differences in sleeping area preference that must be considered: for example, chimpanzees (*Pan troglodytes*) often rest on higher platforms while gorillas (*Gorilla gorilla*) prefer to build ground nests (Earl et al., 2020 [57]). Preferences for the location of nests can also depend on the season; for example, during winter in one study, zoo-housed gorillas spent significantly more time in elevated nests than in floor nests (Lukas et al., 2003 [24]). In a further study of a different great ape, orangutans (*Pongo pygmaeus*) started building nests earlier and for longer than during winter months (Samson & Shumaker, 2015 [58]). Great apes, further, demonstrate a preference for combining multiple types of nesting material such as a combination of hay, paper, blankets, and so forth rather than just one type [59]. Seasonal changes in resting behaviour may necessitate changes in routine to ensure resting and nest-building behaviours are not disturbed by routine husbandry procedures; as well, nesting season necessitates a wide range of different seasonally appropriate and high-quality materials for nest construction.

Many caregivers and institutions work hard to provide appropriate facilities for animals to sleep and nest as they prefer. They will typically provide a variety of nest boxes, shelves, hammocks, branches, and other items for animals to rest and sleep on [6]. However, it is important to note that some primates spend only a portion of overnight hours sleeping. Videan (2006) [60] found that laboratory-housed chimpanzees slept for only 8.8 h per night, which accounts for only half the time that the animals were alone at the facility. Furthermore, these sleep hours were characterized by periods of restlessness and activity throughout the night when there were no animals sleeping. In a further study, Vining et al. (2021) [61] noted that lemurs provided with sleeping quarters enriched with sleeping baskets, multiple choices of ledges, nesting materials, and blankets spent more time sleeping than lemurs in impoverished sleeping conditions with only narrow ledges available to them.

Nocturnal animals who sleep during the day need additional consideration, for the presence of humans, particularly zoo visitors, may disturb their rest. Staff entering enclosures to feed, clean, or care for the animals or low levels of light filtering into their enclosure during routine care may also disrupt their sleep. It is a common practice in many “night houses” designed for the exhibition of nocturnal species that the animals are housed on a reverse night cycle for visitors to witness species-typical active behaviours. However, such lighting cycles mean that animals spend 24 h a day under artificial lighting, which can be harmful, depending on the type of light (Fuller et al., 2016 [62]). Care should be taken to ensure lighting is appropriate for the species, as, for example, using red over blue lights during the artificial “night phase,” as well as ensuring that the light cycles are consistent and appropriate, reflecting seasonal changes where applicable, for the species using technology such as timers.

### 3.2. Challenges and Variations

Key features of captive environments that promote welfare are complexity, novelty, choice, and control (Poole 1998 [63]; Buchanan-Smith 2010 [64]). Variation in the quality and availability of care partly depends on the number of hours the caregivers and veterinarians are at the zoo, research laboratory, or other animal care facility. Humans prefer and benefit from sleeping during the night-time [65], and this results in their working schedules which are to the benefit of the care staff and not necessarily the primates, who could be following an entirely different schedule of daily activities. As an example, while chimpanzees were noted to prefer enrichment related to nesting such as blankets at night in one study, they were also observed interacting with other items such as toys, magazines, and containers at these times (Carner et al., 2013 [66]). Given that animals may be just as active, if not more, within their habitats during the night than they are during the day (Rose & Croft, 2018 [67]), it is worth considering the behavioural opportunities made available to nonhuman primates at night. This includes foraging, exploration, and social enrichment, including times when there are no staff on-site.

Some nonhuman primates, such as small New World species who are sporadically active across a 24-h period, could benefit from having care staff working throughout the night to provide enrichment and foraging opportunities. Of course, the resources are not always available to provide for such a practice. Nevertheless, technology is one way animal care facilities could mitigate the challenges of providing 24/7 access to foraging and enrichment opportunities; for example, simple food puzzles could be placed on a timer and set to become accessible during the night when staff are off-site (Krebs & Watters, 2017 [68]). Such technology provides choice and control during the night-time environment by, firstly, ensuring there are enrichment and foraging opportunities even during the night and, secondly, by giving the animals the choice to decide when they would like to eat within their natural behaviour cycles.

Furthermore, in many zoos, nonhuman primates are often kept within secure overnight facilities during off-hours. This practice can result in behaviours reflective of negative welfare (e.g., Hoff et al., 1994 [69]), particularly in sleeping quarters that are devoid of behavioural opportunities (Vining et al., 2021 [61]). We estimate that primates habitually spend up to 60–70% of their time without a full complement of care staff, while primates housed in back-of-house areas when the zoo is closed will spend on average 16 h inside these areas every day—or a total of 243 days in a year (Brando & Coe, 2022 [5]). Where it is possible, animals can be granted 24/7 access to as many areas of the facility without restrictions to grant the most choice and control over where they want to be and when. Where this is not feasible for safety or other reasons, such as in colder climates, the off-show sleeping quarters should provide as many opportunities for exhibiting both natural active behaviours and natural resting behaviours as possible during out-of-hours times.

Opportunities to provide complexity and variation to primates outside of staff working hours is clearly an area that requires more attention from researchers and animal welfare staff to find the most effective and efficient ways of promoting primate welfare when such housing situations are necessary. Technology is a promising area of research and exploration that certainly warrants increased attention, funding, and study to improve on the behavioural aspects and opportunities for choice and control that can be provided overnight.

### 3.3. Species Differences and Needs

Nonhuman primates in human care comprise a wide variety of species, with an equally wide variety of diversified circadian rhythm patterns (Santini et al., 2015 [70]). Broadly speaking, animals can be categorized in relation to their activity patterns and when during the day or night these activities occur: diurnal (active during the day), nocturnal (night-time), crepuscular (at dawn and dusk), matutinal (dawn and morning), vespertine (dusk and night), and cathemeral (distribution throughout 24 h) are all examples of different 24-h patterns exhibited by primates. Circadian rhythm patterns are not necessarily a strict rule, and some animals may variably display active behaviours outside of their typical waking hours (Tan et al., 2013 [71]). Schedules are flexible and may change throughout an animal’s life as they age (Krebs et al., 2019 [72]). It is important to acknowledge these differences in activity when planning daily routines such as enrichment and feeding schedules, which can differ widely among individuals of even the same species. For instance, Horback et al. (2014) [73] found differences between diurnal and nocturnal activity budgets in an elephant herd housed at the San Diego Zoo, with calves and juveniles initiating most social events not only during the day but also at night-time, which can have positive effects on calf-mother activities. These differences highlight the importance of managing elephants to meet their 24-h behavioural needs (Horback et al., 2014 [73]), and the same philosophy can be applied to other animals, including primates.

In humans, a disruption of the natural circadian rhythm can lead to illness and reduced wellbeing (reviewed in Bechtold et al., 2010 [49]). This is true for other animals as well. For example, rats exposed to constant light or darkness for eight weeks developed depression- and anxiety-like behaviours (Tapia-Osorio et al., 2013 [74]). Although diurnal species of primates are unlikely to be exposed to constant light or dark, nocturnal species, particularly those living in zoos where they are more commonly held, may have their circadian rhythms disrupted by, for example, daytime husbandry procedures, such as staff entering and exiting their enclosure for reprovisioning, needing to be woken up and moved for veterinary and health checks, and other disturbances. Nocturnal animals in zoos are, additionally, commonly kept on reverse light cycles under artificial light to give visitors the opportunity to see active behaviours, which means that if they have access to outdoor areas during their artificial ‘night-time’, they will be exposed to full sunlight. This can overwhelm sensitive visual systems (Erkert, 1989 [75]) and disrupt behavioural patterns; for example, even small amounts of light can decrease activity time and food intake in night monkeys (*Aotus* spp.; Erkert, 1989 [75]). The use of artificial blue light can also influence the health, activity budget, and reproduction of nocturnal animals (Fuller, 2014 [76]). Low levels of additional light may also influence nocturnal primate welfare: observations of wild Azara’s night monkey (*Aotus azari*) suggest that lighting changes across the lunar cycle can alter foraging patterns (Fernandez-Duque, 2003 [77]). As a result of the difficulties of balancing the needs of nocturnal animals with visitors’ desire to engage with these species, nocturnal primates may be confined to smaller and more predominantly indoor enclosures, unlike the housing of their diurnal counterparts.

### 3.4. Safety

Safety is important when promoting animal welfare, and both legal and accreditation safety requirements and procedures are in place to prevent accidents, injuries, and death. These practices include locking animals in indoor holding areas during hours when few or no staff are present, which is a common practice, born out of the legal and institutional concerns about animal escapes, extreme weather, and environmental and physical accidents (BIAZA, 2020 [78]; Brando & Coe, 2022 [5]). Another example of a safety precaution commonly used for captive animals is the practice of splitting social groups into smaller sub-group or single-housed areas for ease of management or to reduce the likelihood of fighting and food monopolization. Although these practices are born from reasonable and real concerns, there may be alternatives worth consideration that result in improved animal welfare. For example, specially designed enclosures and remote weather tracking may make it safer for animals to be given the choice of remaining outside or going inside to escape poor weather, rather than locking animals inside and removing the choice completely. The potential for incidents such as animals escaping, becoming stuck in environmental enrichment, being exposed to adverse weather, or undergoing injuries from social interactions are indeed cause for concern, but they are important during all hours of the day regardless of staff presence. In many institutions, the working schedules of care staff make it impossible for them to observe social interactions, enclosure use, and enrichment use during all working hours as well as outside of hours. The presence of night-time security staff is one consideration facilities could make to allow animals to have access to all areas of the enclosure without compromising safety and legislative restrictions. There is much room for improvement in how care staff balance the practical needs of safety and animal requirements for enriched environments.

## 4. Environmental Enrichment

Environmental enrichment refers to changes to the environment that enhance the physical and psychological wellbeing of captive animals (Newberry, 1995 [79]). By this definition, environmental enrichment includes everything in an animal’s environment that caretakers can change, everything from physical structures and social environment to feeding schedules and the type and novelty of ingredients fed. Much of the environmental enrichment provided to primates, besides the social environment (i.e., living with conspecifics or in mixed-species environments) and physical infrastructure of the enclosure (fixed structures, e.g., climbing frames), relies on the presence of care staff. For example, animal care staff need to prepare, deliver, and clean up after foraging, and provide additional activities and challenges such as puzzles, extra nesting materials, or other items to keep the enrichment program varied and dynamic.

Enrichment should be considered based on its outcomes for animals and scientifically backed with empirical evidence, with systems in place for continuously evaluating and assessing the effectiveness of the enrichment strategies used (Bennett et al., 2018). However, with caregivers present for only 8–10 h per day (with breaks throughout), there are diminished opportunities for supplemental enrichment and other activities, such as behaviour observation, for as much as two thirds of the day. Such time restrictions limit the extent to which caregivers can comprehensively evaluate the impacts of enrichment strategies. As the provision of enrichment is, in many jurisdictions, part of legislative requirements for the care of animals (NRC, 2010 [80]), it is worth considering how caregivers may be able to mitigate this challenge through technology, organisational adjustments, and other interventions.

In the following sections, we will discuss five elements of enrichment: namely, social, cognitive, habitational, sensory and alimentary, and how to address them overnight (Many of the topics that follow are also represented in Table 1).

### 4.1. Feeding

The most appropriate feeding presentation and timing for primates in captivity may differ from what caregivers offer, or from what the facility deems necessary for the species in question. Some species, such as marmosets, start feeding as soon as they wake up in the early morning (e.g., Oftedal & Allen, 1996 [17]; Rylands & de Faria, 1993 [18]; Stevenson & Rylands, 1988 [19]). However, their diet might not arrive until many hours after dawn when care staff are on site and able to deliver it. Having a more predictable environment and greater overall access to food can reduce the negative effects of unpredictable feeding times that can arise due to changing caregiver routines (Bloomsmith & Lambeth 1995 [81], Brando 2009 [82]; Krebs & Watters 2016 [83]; Waitt & Buchanan-Smith 2001 [84]).

To facilitate more reliable and predictable feeding opportunities in the hours when staff are absent, timers, ice blocks and self-operated food boxes can be used for food distribution (see Sommerfeld et al., 2005 [20] for an example with lemurs). Where possible, staff working schedules could be adjusted to accommodate the species they care for—for example, with staff coming on site to feed animals earlier and leaving earlier in the day, or using a “shift” system, where different members of staff come on site at different times during a 24-h period to ensure optimal staff wellbeing balanced with species-specific animal care.

### 4.2. Cognitive

Sleep is not the only activity that diurnal primates are engaged in at night, nor is it a matter of them sleeping from the moment staff leave until they return the next day. These animals forage, play, and socialize in the hours before and after caregivers are present and may benefit from the provision of occupational and cognitive enrichment, such as touch screens (Calapai et al., 2022 [85]; Perdue et al., 2012 [86]) or puzzle toys (Calapai et al., 2017 [87]; Clark et al., 2019 [88]; Reinhardt & Rodgers, 1997 [89]), during these hours. Providing opportunities that increase the time spent exploring, foraging for, processing, and consuming food is an important form of cognitive and occupational environmental enrichment that frequently overlaps with feeding enrichment goals. However, such enrichment items still occupy only a small number of the hours that animals may spend unattended throughout the evening and night. In the absence of automated feeders and care staff, these enrichment items are unlikely to be delivered in the late evening or early morning hours when primates would forage in their natural habitat, or at night, for nocturnal species.

Much of the environmental enrichment that requires time and encourages solving tasks can be mounted on top or on the sides of the cages because placing these activities on the exterior with more difficult access makes it harder to forage for food. Opportunities for many different animals to access the games at the same time can be increased when multiple spots are provided; this reduces or in some cases avoids entirely the risk of more dominant animals having monopoly over the devices, which is an important consideration when socially housing animals during hours when there is a lack of supervision. There should always be enough provisions for all to prevent fighting or individual animals monopolizing resources. This is particularly important during off-hours when care staff are not present to ensure the less dominant animals are still able to access food through supplementary feeding or other methods. For example, macaques are commonly housed in compatible pairs in research contexts, and access to food and enrichment for both the dominant and the subordinate individual must be assured.

There are many examples of low-budget enrichment options which can be used to provide cognitive challenges during the off-hours. These include puzzles with holes that make it harder to forage or require tools to access rewards (Padrell et al., 2022 [90]; Yamanashi et al., 2016 [91]), or a pipe securely mounted to the outside of the mesh with food items they must access using their fingers or tools, hanging up frozen treats which slowly defrost, or fake grass platforms with small seeds hung on the outside of the cages. Another example of cognitive and occupational environmental enrichment is to provide different sleeping areas and nesting substrates, and providing enough of these to offer individual animals a choice and opportunity to construct their own sleeping places during the night with the materials they prefer.

Other types of activities could include timers that release or propel food at a set time, into a pile of substrate, for example. Touch screens are much more expensive but offer opportunities to play games (Calapai et al., 2022 [85]; Fagot & Bonté, 2010 [30]; Perdue et al., 2012 [86]). By narrowing the gap between different disciplines, programmers, and animal caregivers, it could be possible to program these machines and other types of animal-controlled technology (Washburn, 2015 [92]) to respond to request food or desired items, doors to be opened or closed: the technology is there, and so are our and others’ ideas (see Coe & Hoy, 2020 [93] for a more in-depth discussion of the topic). Training can be used to provide animals with more autonomy during the night using cognitive enrichment including such devices (Cabrero-Moreno et al., 2022 [94]). There is even the potential for such software to be used to provide training to animals during off-hours (O’Leary et al., 2018 [95]), as was demonstrated with the experimental “Mymou” program which allowed macaques opportunities to develop and learn new behaviours without human intervention (Butler & Kennerly, 2019 [96]). While much of these experimental enrichment designs were implemented and tested as daytime behavioural strategies, there are ample opportunities to utilise these ideas over night as well. There are a great number of studies discussing the cognitive enrichment of primates during the day (Clark et al., 2019 [88]; Yamanashi and Hayashi, 2011 [97]), but there is a need for further research on how to best meet the cognitive needs of primates during off hours and at night and the benefits of doing so. This is also particularly needed for primates housed in research laboratories and subjected to spatial and social constraints.

### 4.3. Social

For some primates, particularly smaller species such as tamarin and marmosets, family groups may choose their sleeping sites and partners (see Caine et al., 1992 [56]; Cui et al. 2006 [98]; Vessey, 1973 [99]). This leaves individuals open to meet their social enrichment needs overnight, provided that adequate sleeping sites are available to choose from. Great apes, in contrast, are sometimes separated into individual sleeping arrangements depending on the facility. While such separation may be necessary to keep all individuals in a group safe, reduce wounding, and promote individual welfare, the practice reduces overnight social choices. Some facilities reduce potential conflicts by separating certain individuals when necessary; for example, housing high-ranking males or older individuals with special needs overnight separately, and leaving doors open to allow choice among the remaining individuals in the group. It is important to monitor the behaviour of the colony during the night in the case of social expulsion, as the expelled individual could be prevented from sleeping in the nest with the others (personal observation Augusto Vitale) or wounded when shut in smaller overnight quarters where it is not as easy to get away from aggressors (personal communication Max Norman). Alternative nesting sites should be provided in these cases (De Filippis et al., 2009 [100]), as well as enough space and escape routes for animals to get away if they need to.

### 4.4. Physical Habitat

Another important consideration is the physical environment that primates occupy during off hours. Most facilities include secure indoor holding facilities, and several factors influence the amount of time that animals must spend indoors. Laws and regulations, staff shortages, maintenance of the enclosure, weather, the presence of infants are all factors to consider in deciding whether individual animals are to be granted access to indoor and/or outdoor areas at any given time.

There is evidence that a combination of indoor and outdoor housing options improves welfare for a variety of primate species (e.g., Castro et al., 2003 [27]; Ruivo et al., 2015 [21]). In zoos and other facilities, open access is likely to be important for species that are active during off-hours but nonetheless should not be discounted for species that predominantly rest and sleep during that time. Locking or keeping animals inside can result in behaviours associated with negative welfare; for instance, Hoff and co-authors noted increased aggression between mother and infant gorillas while they were inside, and the researchers also found more self-directed behaviours in infants and more auto-grooming in mothers while housed inside (Hoff et al., 1994 [69]). The size of the social group may be important when animals are shut into back-of-house areas overnight. In a study of elephants, a higher percentage of time spent locked inside was associated with increased indicators of stress (Carlstead et al., 2019 [101]), potentially due to the compounding effects of increased stocking densities overnight. The same is likely to be true for nonhuman primates who are housed using similar practices where the group is forced into proximity during the night. Locking up inside could paradoxically increase the risk of injury and wounding rather than increase safety. Giving animals the option to move off the exhibit and into back-of-house areas when they choose is also important. For example, one study of common marmosets found that the animals had a strong preference for being outdoors during the day despite access to a large and highly enriched indoor room, which appeared less attractive to the marmosets during daytime hours (Pines et al., 2007 [102]). Although indoor areas may be heavily enriched, locking animals inside during off-hours could represent a welfare challenge when they would choose to be elsewhere given the option. While some zoos may house primate species that live in colder climates, such as Japanese macaques (*Macaca fuscata*), other primates originally live in sub-tropical climates. Species from a different climate or ones prone to injury in these conditions need special care. Heat lamps, vegetation, shelters, and other features in the in- and outdoor areas can be utilised to create different microclimates based on their current temperature, humidity, and other environmental parameters.

### 4.5. Sensory

Sensory enrichment includes the sights, sounds, scents, and textures of the environment. Primates with both indoor and outdoor access can experience all these sensations, including stimuli such as the zoo waking up in the mornings or closing at night. For animals with limited access to the outdoors, such as nocturnal animals in a night house or individuals kept indoors or in a research laboratory with no outdoor facilities, supplemental sensory enrichment can simulate a more natural environment. This can be in the form of vegetation, different natural and artificial materials that can be fixed or flexible, different tactile experiences, objects of different sizes, manipulanda in the form of puzzle feeders, and acoustic stimulation through sounds. Foraging for insects with slow-release devices or items in which insects can live will increase foraging time.

Technology can grant further control over environmental parameters (e.g., additional light and localized heat for marmosets: Buchanan-Smith & Badihi, 2012 [29]). For example, indoor fluorescent lighting may be operated by automatic time switches adjusted seasonally to mimic the natural changes in day length in the species’ habitat. This could also be used to give primates the option to adjust other parameters such as temperature and humidity to levels that are thermally comfortable for good-quality sleep (Ayuso et al., 2023). This benefits primate welfare by affording opportunities to operate their environment independently from human presence to a certain extent and exercise some control and choice over their habitat.

## 5. Habitat Management, Monitoring, and the Use of Technologies

The essence of care for any animal is habitat management [103]. Planning and designing environments that incorporate animals’ needs and preferences are of particular interest to professionals working with them, for habitat management aims to reduce the animal’s dependency on humans. Habitat management in captivity provides for species-specific needs and should be modified to reflect and respond to individual needs and preferences over time and with consideration of the off hours, including microhabitats.

It is important to foster environments that contribute to the mental and physical wellbeing of animals through built-in functional enrichment (Coe, 1989 [104]; 1996 [105]; 2003 [106]; 2011 [107]; Coe and Hoy, 2020 [93]). Functional infrastructure can include platforms, shelves, larger branches to huddle, rest, and sleep on, or puzzle feeders and timers that can deliver food treats and smaller enrichment items. The use of computers to deliver food rewards or games and problem-solving puzzles is not new. Markowitz (1982) [108], for example, described how he taught a mandrill to use a simple computer to play tic-tac-toe with visitors and primates to exchange tokens for food items. A similar concept could be used to provide access to food or toys when staff are not available during off-hours. For primates used in laboratory research, these solutions would have to be balanced with the recurrent need for the animals to perform experimental tasks. Creative and different approaches, such as the research cubicles at the Edinburgh Zoo in Scotland and the Laboratoire de psychologie cognitive in France, which allow animals to come and go and participate at their choice, should be explored, as well as the opportunity to use technology as part of animal training procedures (O’Leary et al., 2018 [95]; Butler & Kennerly, 2019 [96]).

Choice and control are particularly important areas of animal welfare and wellbeing to consider and integrate into habitat design. A large platform that facilitates social primates sleeping together under a heat lamp, as well as space on the platform to be away from the heat lamp, and the option to switch the lamp off with an animal-centred technology such as a button or switch are all examples of providing choice and control for the animals and fulfilling the needs mentioned in Table 1. In contrast, a smaller platform without heat lamps will affect the social group and reduce the number of possibilities for the social primates to sleep together or choose from different micro-climates. Providing multiple options is encouraged to allow more choice to the animals. There are a multitude of other technologies which can be used to give animals control over their environments. The use of motion detectors or pedal-driven mechanisms can be provided to chimpanzees and other primates (Coe, 2006 [31]). Marmosets can touch sensors to control supplementary light (Buchanan-Smith & Badihi, 2012 [29]). Interactive computer screens have been used for baboons and chimpanzees to play games or request food (Fagot & Bonté, 2010 [30]) (Savage-Rumbaugh et al., 1986 [109]). Recently Kinect 3D, an add-on for the Xbox 360 gaming console, has been used to create an interface for orangutans at the Melbourne Zoo. This system provided a natural user interface that allowed the orangutans to interact with the games and activities broadcast onto the floor without an intermediary device, such as a controller, so was safe for use with this and other large species (Carter et al., 2021 [110]). The program turns the floor of an enclosure into a touch screen and can be activated by various body parts as well as touches with blankets, which is the often-beloved item orangutans like to hold. All six of Melbourne Zoo’s orangutans are currently involved in a study of their responses to interactive games, including puzzles and colour discrimination, as well as their individual differences with these activities (Carter et al., 2021 [110]).

All these different activities, enrichment, types of housing and other provisions can promote positive and optimal welfare. However, it is important to have an evidence-based approach to animal welfare. Scientific monitoring is a long-standing practice to assess the welfare of zoo-housed animals (Kleiman, 1992 [111]) and it is as necessary outside of regular working hours and overnight as it is during the day. Video cameras and recording systems make behavioural observation of primates during off hours feasible, and many different technologies, including bioacoustics and passive acoustic monitoring, are available to monitor and assess animal welfare without the need for human presence (Whitham & Miller, 2016 [112]). Acoustic monitoring could be used, for example, to assess the vocalisations of primates at night that may indicate fragmented sleep, as was studied in orangutans (Samson et al., 2014 [113]). Digital tracking is a newer form of behavioural monitoring with potential applications for overnight assessment of animal behaviour, using pose-tracking technology to detect the different behavioural states experienced overnight (Hayden et al., 2021 [114]; Knaebe et al., 2022 [115]). An overview of the available technologies and their applications to off-hour care is in Table 2. Analysis of such footage and data, which can be reviewed even off-site and at any time, will allow animal caretakers to better understand the nocturnal activities of animals including sleep, use of enrichment, play, and other behaviours.

Habitat management in combination with a wide variety of technologies can provide foraging activities, including more complex enrichment types, and provide options to monitor and assess off-hour animal activity.

## 6. Conclusions

Promoting primate welfare outside regular working hours is an important task for animal care staff. The combination of optimal habitat design with a wide variety of technologies, which includes environmental complexity of indoor and outdoor areas to reduce dependency on human care, can facilitate more choice and control, increasing agency and access to enriching activities such as puzzle feeders on timers and interactive foraging panels. All of these and the other strategies play a key role in the promotion of positive animal welfare and should incorporate the monitoring and assessment of animal activity during off-hours so evidence-based decisions can be achieved regarding the night-time care of nonhuman primates.

The impact on the welfare of primates closed in smaller areas for prolonged time overnight needs to be mitigated with prompt action and made subject to further investigation in the peer-reviewed literature. Suitable solutions for spatial restrictions through habitat development must be found, and recommendations on the requirements and care of primates during night hours should be reflected in the best practice guidelines for all taxa. Primate care and welfare should be considered and enhanced, considering their needs and preferences from a 24/7-across-lifespan perspective, including all hours when less or no staff are present at the facility.

## Figures and Tables

**Table 1:** This table highlights 14 welfare criteria with examples of what to consider when promoting primate welfare when staff is absent. Adapted from Brando and Buchanan-Smith 2018; the first two columns are directly from this paper, with our commentary in the third column.

Welfare Principles		Welfare Criteria	Examples of Aspects to Consider When Promoting Positive Welfare When Staff Is Absent
Good feeding: Are animals properly fed and supplied with water?	1	Absence of prolonged hunger (i.e., mimic natural feeding intervals). The other end of the spectrum (obesity) should also be considered.	Provide access to food, gum, and bark to gnaw on from sunrise until late afternoon to sunset (e.g., Oftedal & Allen, 1996 [17]; Rylands, 1993 [18]; Stevenson & Rylands, 1988 [19]). Timers and self-operated food boxes can be used for food distribution in off hours (lemurs: Sommerfeld et al., 2005 [20]).
	2	Access to appropriate food and species-typical foraging opportunities (i.e., they should have a nutritionally suitable and appropriate diet and delivery).	Depending on the species, provide leaves, gum, bark to gnaw on, and insects during off-hours. Food can be left in vegetation, in an overhead cover to drop down, or on the exhibit floor to encourage hunting for small, hand-held pieces. Larger pieces can be left in more challenging positions to encourage upside-down hanging and other types of locomotion and physical activity. A wide variety of different food enrichment presentations and locations should be provided (with attention to social influences).
	3	Absence of prolonged thirst (i.e., they should have a sufficient and accessible water supply).	Prolonged thirst might be experienced after many hours without access to water. Provide clean water in indoor and outdoor areas 24/7, in multiple places to reduce competition or monopoly (Ruivo et al., 2015 [21]). Water systems that refresh automatically can also be used to ensure constant access to fresh water.
Good housing: Are animals properly housed?	4	Animals should have comfort when they are (socially) resting, i.e., physically comfortable and relaxed (e.g., not always vigilant) when resting and sleeping.	Provide different resting and sleeping platforms in various places—shaded, sunny, and covered—as well as nesting boxes and hammocks, all large enough to facilitate social resting and sleeping. Provide cover to reduce vigilance and increase sleeping, resting and relaxation; a variety should be provided indoors and outdoors, possibly closer to the public as well as in concealed and quieter areas for primates who sleep during the day. Fading light for sunrise and sunset allow animals to wake slowly and settle for the night (Cunha et al., 2006 [22]; Ruivo et al., 2015 [21]). Great apes tend to construct elaborate nests in the wild, high in the trees and out of sight of predators (Goodall, 1962 [23]). Zoo-housed gorillas were observed to spend significantly more time in elevated nests than in floor nests, especially in winter (Lukas et al., 2003 [24]). Options are a variety of shelves, hammocks, nest boxes, crates, and ropes; the back of house must be made safe and accessible.
	5	Animals should have thermal comfort, i.e., they should neither be too hot nor too cold and have thermal zones to choose from.	Heat lamps should be installed in indoor and outdoor facilities. Multiple lamps should be in a variety of places, concealed for resting and sleeping, or above the animal’s lookout, which may be vegetation-covered. Hiding places and shelters away from wind, rain, and cold drafts should be provided. If open access to indoor and outdoor areas is provided, a covered door is preferred to maintain warmer indoor areas during cold spells. Indoor humidity and weather should be regularly checked, and a ventilation system should be in place to ensure healthy airflow (e.g., De la Fuente et al., 2014 [25]; Ruivo et al., 2015 [21]).
	6	Animals should have enough space to be able to move around freely and naturally (e.g., leaping distances, orienting substrates, etc.), and indoor-outdoor space restrictions.	The presence of relevant incentives to engage in species-appropriate behaviours increases primate wellbeing (Chang et al., 1999 [26]). Indoor and outdoor spaces should preferably be available. Sufficient height should be provided for arboreal species (at least 3.5–4 m; Ruivo et al., 2015 [21]). Different structures, such as a mesh and natural branching, should be used to increase useable surface areas, in both indoor and outdoor areas (e.g., Brando & Coe, 2022 [5]; Castro et al., 2003 [27]; Ruivo et al., 2015 [21]) with perches at varying heights and angles (for laboratory primates see Rennie & Buchanan-Smith, 2006 [28]; Rennie & Buchanan-Smith, 2011)
	7	Animals should have perceived control (e.g., complex enclosure giving them choice over what and when they do things).	Different areas, thermal zones, light levels, and concealed/hiding places should be provided. There should be a wide variety of environmental enrichment opportunities. Technology can be used to increase the options for control over environmental parameters (additional light and localized heat for marmosets: Buchanan-Smith & Badihi, 2012 [29]). Computer screens can be used to request food (baboons: Fagot & Bonté, 2010 [30]). The use of (infrared) motion detectors or lever-activated enrichment can be provided to chimpanzees and other primates (Coe, 2006 [31]).
Good health; Are the animals healthy?	8	Animals should be free of major injuries, e.g., skin damage and locomotory disorders.	The environment should be safe from sharp edges and other items that could harm the animals. Environmental enrichment should be checked for safety. Animals should not be caught in the nest boxes, awake or when sleeping or resting.
	9	Animals should be free from disease, i.e., meeting appropriate standards of hygiene and care.	Appropriate standards of hygiene without compromising other areas of animal care, e.g., use of bio floors in indoor enclosures (Leinwand et al., 2021 [32]).
	10	Animals should not suffer pain induced by inappropriate management, handling, catching, or transport.	Positive reinforcement training should be used as a way of reducing stress related to regular husbandry procedures, and experimental testing, including shifting between different areas (Schapiro et al., 2003 [33]),and shifting to indoor areas for the night.
	11	Animals should be treated well in all situations (i.e., care staff should promote good human-animal relationships, with the animal’s perspective as the focus).	Good human-animal relationships should be encouraged. Animals should be trained to participate in their daily care, e.g., shifting, weighing, body inspection, coming close to the caregiver (McKinley et al., 2003 [34]; Prescott & Buchanan-Smith, 2016 [35]), including shifting to indoor areas for the night, and participate in experimental procedures (for common marmosets see Manciocco et al., 2009 [36]).
Appropriate behavior: Does the behavior of the animals reflect optimized emotional states?	12	Animals should be able to express normal, non-harmful social behaviours (e.g., grooming).	Social primates should be housed in social groups, preferably as an (extended) family. Appropriate environments (e.g., hammocks, large horizontal branches) and stable groups allow for affiliative behaviours such as huddling, resting, sleeping, grooming, food sharing, and playing together (e.g., Digby & Barreto, 1993 [37]; Tardif et al., 1984 [38]; Heymann, 1995 [39]; Smith et al., 2007 [40]; Leonardi et al., 2010 [41]). Generally, in research laboratories, primates are still housed in compatible pairs due to space limitations (e.g., DiVincenti & Wyatt, 2011 [42]; Hannibal et al., 2017 [43])
	13	Animals should be able to express other normal behaviours, i.e., it should be possible to express species-specific natural behaviours, e.g., burrowing, exploring, scent-marking.	Primates should be able to perform natural behaviours, such as foraging and gum gouging, insect hunting, or solving novel food tasks in off-hours. Flexible and fixed structures should be provided to allow for a varied pattern of locomotion with high points for wider views and for sunning in the morning before the staff arrives (e.g., Hardie et al., 1993 [44]; McGrew et al., 1986 [45]; Molzen & French, 1987 [46]).
	14	Negative emotions such as fear, distress, frustration or boredom/apathy should be avoided, whereas positive emotions such as security or contentment should be promoted.	Good social groups are important to primate welfare for playing, grooming, resting, sleeping and interacting together. A varied and complex environment with perceived control and extensive environmental enrichment should be provided to allow for activity, opportunities to play, and feeling sheltered and secure (Young, 2013 [47]; McKinley et al., 2003 [34]; Ruivo et al., 2015 [21]). For example, a playroom could be created to be routinely visited by the primates housed in the laboratory.

**Table 2:** Overview of monitoring technologies available and their applications to the off-hours care of primates.

Technology	References	Applications to Off-Hours Care of Primates
Video cameras and recording systems	Hansen et al., 2018 [116]	CCTV can be installed with wide coverage of off-hours areas of the enclosure. These video streams can be connected to livestreams which can be remotely accessed or saved as recordings to be reviewed later.
Bioacoustics	Whitham & Miller, 2016 [112]	Depending on the species in question, bioacoustics can be used to pick up on vocalisations indicative of animal welfare states overnight as well as detect periods when animals are awake and vocalising.
Digital tracking (i.e, pose-tracking)	Ballesta et al., 2014 [117]; Hayden et al., 2021 [114]; Knaebe et al., 2022 [115]	Automated detection of behavioural states overnight using pose-tracking could be used to create assessments of how much time primates engage in particular behaviours, e.g., sleeping, without need for review by a human observer, saving time and resources to develop in-depth overnight activity budgets

## Data Availability

Data sharing not applicable. No new data were created or analyzed in this study. Data sharing is not applicable to this article.
